# End of life care for people with dementia: The views of health professionals, social care service managers and frontline staff on key requirements for good practice

**DOI:** 10.1371/journal.pone.0179355

**Published:** 2017-06-16

**Authors:** Richard Philip Lee, Claire Bamford, Marie Poole, Emma McLellan, Catherine Exley, Louise Robinson

**Affiliations:** 1 Institute of Health & Society, Newcastle University, Newcastle-upon-Tyne, United Kingdom; 2 Faculty of Health & Life Sciences, Northumbria University, Newcastle-upon-Tyne, United Kingdom; University of Glasgow, UNITED KINGDOM

## Abstract

**Background:**

Evidence consistently shows that people with advanced dementia experience suboptimal end of life care compared to those with cancer; with increased hospitalisation, inadequate pain control and fewer palliative care interventions. Understanding the views of those service managers and frontline staff who organise and provide care is crucial in order to develop better end of life care for people with dementia.

**Methods and findings:**

Qualitative interviews and focus groups were conducted from 2013 to 2015 with 33 service managers and 54 staff involved in frontline care, including doctors, nurses, nursing and care home managers, service development leads, senior managers/directors, care assistants and senior care assistants/team leads. All were audio recorded and transcribed verbatim. Participants represented a diverse range of service types and occupation. Transcripts were subject to coding and thematic analysis in data meetings. Analysis of the data led to the development of seven key themes: Recognising end of life (EOL) and tools to support end of life care (EOLC), Communicating with families about EOL, Collaborative working, Continuity of care, Ensuring comfort at EOL, Supporting families, Developing and supporting staff. Each is discussed in detail and comprise individual and collective views on approaches to good end of life care for people with dementia.

**Conclusions:**

The significant challenges of providing good end of life care for people with dementia requires that different forms of expertise should be recognised and used; including the skills and knowledge of care assistants. Successfully engaging with people with dementia and family members and helping them to recognise the dying trajectory requires a supportive integration of emotional and technical expertise. The study strengthens the existing evidence base in this area and will be used with a related set of studies (on the views of other stakeholders and observations and interviews conducted in four services) to develop an evidence-based intervention.

## Introduction

By 2050 it is estimated that at least 131 million people worldwide will live with dementia [1]. In England and Wales, dementia is the leading cause of death with 11.6% of deaths in 2015 attributable to dementia [2]. A significant proportion of the total costs of dementia care occur during the last year of life [3, 4]. Evidence consistently shows that people with advanced dementia experience suboptimal end of life care (EOLC) compared to those with cancer, with increased hospitalisation, inadequate pain control and fewer palliative care interventions [5–12]. People with dementia are also likely to be living, and dying, with significant co-morbidity [13]. In England and Wales their care–in care homes, own dwellings and in hospital–is provided within a mixed public and private health and social care system. The health system has increasingly shifted towards a (not uncontroversial) model of commissioning and contracting led by clinical commissioning groups, in an attempt to increase care skills and align general practitioners to the wider health system [14, 15]. At the same time social care budgets, managed by local authorities, have been subject to increased pressure with overall expenditure falling against increased demand driven by demographics [16].

Meeting these complex challenges requires an evidence base that can help to identify how good end of life care for people with dementia can be delivered and the form it should take. In the UK, current National Institute for Health and Care Excellence (NICE) guidance on dementia supports a palliative approach for EOLC despite a lack of high quality empirical evidence [17]. Recently national and international policy initiatives aimed at improving the quality of EOLC for older people in general, as well as those with dementia have been introduced [18–22]. Recommendations for what constitutes optimal standards for EOLC for people with dementia are also available [23] but have been largely developed through expert professional consensus in the absence of high quality, empirical research [1]. In addition to concerns around the quality of EOLC in dementia, issues exist around continuity of care and preferred place of care, especially at end of life (EOL). Very few people with dementia die at home or in hospice care; the majority die in care homes and around a third in acute hospitals [24, 25].

In order to contribute to the emerging evidence base, in this paper we explore the views of health professionals, service managers and frontline care staff on key aspects of good EOLC for people with dementia. We have focused on care homes, supported living and hospice settings, but have necessarily sought the views of a range of participants from across health and social care. Crucial to the development of any quality initiatives to improve EOLC is an understanding of the views of key stakeholders involved in providing and receiving such care. In the UK, national expert views have been considered [26], but of equal importance are the views of those who are directly responsible for dementia care delivery i.e. service managers and frontline care staff. Studies conducted with these professional groups have suggested that poorly developed teamwork and leadership in dementia care have a major impact on the quality of care provision in nursing homes [27, 28]. An evidence review [28] has shown that higher levels of comfort are experienced by people with dementia at EOL when staff are working with dementia specific approaches and have specialist support and education [29–31], although inequalities remain between EOLC provision for those with dementia compared to people with other conditions [8, 32–37]. Studies exploring the communicative and emotional aspects of EOLC in dementia [38–42] suggest attention should be focused on encouraging space for communication between key groups (staff, family members and people with dementia) in nursing homes, to address possible conflict [38]. Bassal and colleagues examined the factors influencing the ‘emotional exhaustion’ of staff providing EOLC in dementia, and drew attention to the potential negative consequences of suppressing emotions [39]. It has also been suggested that the positive, context specific use of ‘detachment’ (staff distancing themselves emotionally from residents when carrying out care work) and ‘engagement’ (staff engaging emotionally during care) by healthcare assistants providing in-patient dementia care complicates classifications of good (and bad) care [40].

One study specifically sought to understand the factors that professional, and family, carers considered contributed to a good quality death for people with dementia [41]. Three key elements of good EOLC for people with dementia were identified: meeting physical care needs, going beyond task-focused care and planning and communicating with the family. For professional carers, these elements encompassed appropriate pain management, recognition that the actions of people with dementia can be intentional and meaningful [43], and uncertainty over the appropriate timing of advance care planning conversations (advance care planning involves discussions about a person’s future care while they still have capacity, which are often formally documented) [44].

### Aim

The aim of this paper is to explore the views of service managers and frontline care staff on key aspects of good EOLC for people with dementia in order to contribute to the evidence base and to highlight key considerations for any future intervention intended to support professionals to provide better quality care. EOLC is understood as encompassing early discussions about end of life care to final days. We present in detail seven key themes, developed from semi-structured interviews and focus groups with 87 service managers and frontline staff, as a further contribution to the (currently sparse) evidence base.

## Methods

Ethical approval for the research was granted by: UK Health Research Authority NRES Committee North East—Newcastle & North Tyneside 1 (13/NE/0335). In order to understand the perspectives of a range of service managers and frontline staff providing care (Table 1), the first stage of data collection involved semi-structured interviews with service managers from a range of services across England, including some participants who did not directly manage a service, but were responsible for the conduct or co-ordination of aspects of EOLC for people with dementia. Our definition of EOLC included early discussions about dying, through to final days and subsequent care for relatives. The study was focused on community care (namely in care homes, hospices and dwellings). Thirty three service managers were interviewed; our sample comprised a wide range of professional staff including doctors (n = 4) and nurses (n = 7); nursing and care home managers (n = 12); service development leads (n = 5), and senior managers/directors (n = 5). Interviews last between 27mins and 1h 03mins. The topic guide focused on: details of service and relationships with other services; key components of good EOLC for people with dementia; how this differs from/is similar to EOLC in other conditions; perceived value of existing frameworks and approaches. Service managers were recruited through a combination of recommendations from national experts [26], identification of services from the wider study team and snowballing. Following the interviews with service managers, we purposively selected eight sites across England for focus groups with frontline staff, making use of contacts established in earlier phases of the research programme. Services were selected to ensure a range of service type and location and were identified as being good or standard practice. They included two care homes, two specialist elderly mentally infirm (EMI) homes, two hospices and two services providing a range of services including group living homes and home care. Three were urban, three suburban, one rural and one semi-rural. A total of 54 staff took part in ten focus groups (two focus groups were conducted in two services). Participants in the focus groups included care assistants (n = 18); senior care assistants/team leads (n = 7); nurses (n = 17); doctors (n = 1), service development leads (n = 4) and managers (n = 7). Focus groups lasted between 48mins and 1h 48mins. Staff involved in end of life care for people with dementia were deemed eligible for participation. The topic guide focused on: successful (and less successful) examples of EOLC for people with dementia; key components of good practice; how this differs from/is similar to EOLC in other conditions; perceived value of existing frameworks and approaches. The composition of focus groups included care assistants, care home nurses, hospice clinical staff and senior care home managers, though this varied with each focus group.

**Table 1:** Participants in interviews and focus groups.

	Interviews	Focus groups	
	Service managers	Frontline staff	Total
Residential nursing/care homes	12	16	28
Specialist EMI services	3	15	18
Supported living	5	10	15
Primary care	4	0	4
Specialist palliative care services	9	13	22
Total	**33**	**54**	**87**

All focus groups and interviews (conducted by EL, CB, MP and EM) were audio-recorded and transcribed verbatim. The transcripts of the interviews and focus groups were coded using a frame developed from open codes and their discussion in data meetings, involving RL, CB, MP, EM and CE. Our discussion in the data meetings focused on the integrity of codes and the coherency of themes. Codes were then grouped thematically, with continuous iterations between original transcripts and the developing themes [45, 46]. Data saturation was reached as no new, significant themes emerged during the analysis.

## Results

Providing good EOLC for people with dementia is highly demanding and complex work. While the general issues discussed by service managers and frontline staff were similar, the ways in which they described their personal experiences and views of EOLC differed. Service managers tended to talk at a more abstract level while frontline staff often spoke in detail about specific cases of EOLC. Our analysis identified seven key themes:

- recognising EOL and tools to support EOLC
- communicating with families about EOL
- collaborative working
- continuity of care
- ensuring comfort at EOL
- supporting families
- developing and supporting staff

The themes are described in detail below with supporting data; where relevant, differences in emphasis and perspective between service managers and frontline care staff, and also between staff from different types of services, are highlighted and discussed.

### Recognising EOL and tools and processes to support EOLC

Service managers and frontline staff often described recognition of EOL as a combination of symptoms and behaviours or a specific “key moment”. Services with a focus on person-centred care discussed how subtle changes indicated that a person with dementia may be approaching the EOL; staff infrequently described sudden deaths, suggesting most people with dementia showed discernible changes. Identifying such changes required a considerable degree of skilled interpretation given the significant difficulties in establishing verbal communication. Staff without a relationship with a person with dementia could find it difficult to identify subtle changes particular to that person;

You see a change, but you can’t go on the phone and say to the doctor, “Well she’s changed” because there isn’t a word for it […] it’s like a gut feeling for me. [Staff focus group, manager, supported living service]

…with things like dementia and Alzheimer, it’s really hard to recognise that people are dying. [Staff focus group, senior care assistant/team lead, specialist palliative care service]

Some services had adopted a proactive and systematic approach to identifying people approaching the EOL, using tools like the Gold Standards Framework (GSF) [47] or in-house approaches such as team meetings. However, staff in one service working towards GSF accreditation found the process of categorising people with dementia at odds with their person-centred ethos and were uncertain it helped identify people approaching EOL. In contrast, other staff suggested that a systematic process of identifying care home residents towards the EOL improved outcomes, including hospital avoidance:

[We have a] monthly meeting to look at the register to see where the residents are […] as they deteriorate or as they go through the trajectory phases of dying then we discuss it as a team with the Macmillan group. [Interview, service manager, residential/nursing home]

[GP was saying] the difference it has made in that nowhere near the number of patients from that care home are being whipped into hospital. [Staff focus group, nurse, specialist palliative care service]

Notwithstanding a proactive, systematic approach to the recognition of people with dementia approaching EOL prompted: review of plans; use of EOL pathways or emergency health care plans; medication review, more regular GP review, discussions about access to anticipatory medicines and preparing families for anticipated changes. Whilst the Liverpool Care Pathway (LCP) (a widely used clinical protocol for care in the last days of life) was phased out in England in 2014 [21, 48], local versions of this tool continued to be used.

[The LCP] it’s not just a yes/no tick box, it makes you do something […] we are still at the moment using the LCP as a tool and I’ve found it really good. [Staff focus group, nurse, residential/nursing home 2]

Other tools or processes–such as advanced care planning (ACP), emergency health care plans and do not attempt resuscitation (DNACPR) forms–were also identified as useful to clarify wishes and initiate sensitive conversations. A number of issues were identified concerning ACP. However, managers recognised the difficulties of raising the issue of ACP shortly after dementia diagnosis, both on a practical level (in terms of timing and resources) and as preferences might change. Views on who should be responsible for ACP varied. While hospice and palliative care staff had expertise, they felt that their role was in supporting other professionals to take the lead on ACP discussions. Care home managers tried to mitigate these difficulties by getting to know residents and using informal and opportunistic approaches to capture preferences and acknowledged that they sometimes had to ‘second guess’ preferences.

…I think we take the cues from the residents and from the families as well but sometimes it can be just simple questions that they’re able to answer like ‘How do you feel about going into hospital?’ Most people have got a reaction to that. [Interview, service manager, residential/nursing home]

However, concerns were raised by specialist external staff (e.g. hospice staff, community matron) about the quality of ACP in care homes and the willingness and ability of GPs to engage in discussions around ACP and DNACPR was questioned: The issue of DNACPR was particularly challenging although some care homes reported a proactive approach to this with documentation being completed on admission.

I think nursing homes do some advance care planning […] I fear it's a bit tick-boxy. […] [Interview, doctor, specialist palliative care service]

I think there’s a lot of work to do with getting to the GPs because they don’t really understand what they’re doing. [Staff focus group, service development lead, supported living service 2]

The ‘do not resuscitate forms’, they used to be always done quite late on, it would be touched on possibly at admission, but […] we’re getting them activated straight away [Staff focus group, nurse, residential/nursing home 2]

In summary recognition of EOL in people with dementia required both technical skills and personal, ongoing knowledge observing and interpreting signs of deterioration and systematic approaches to acting on this information. Although there were practical issues with the use of generic EOL tools for people with dementia, this generally prompted a more proactive and structured approach.

### Communicating with families about EOL

Ensuring that family members were aware of their relative approach EOL, and were involved in key decision making was consistently identified as important A distinction was drawn between abstract discussions about EOLC (when a person with dementia was in a stable condition) and more specific discussions prompted by a deterioration. Abstract discussions facilitated discussion of dementia as a terminal illness and introduced future care planning. Staff described a gentle approach to these conversations, typically beginning with the family’s perception of their relative’s health, although confidence and experience in initiating such discussion varied between individual staff. Staff confidence and skills in initiating discussions varied; some acknowledged avoiding such conversations, others felt able to approach them:

…gradually get into it, whether it’s over a course of a couple of hours whilst they’re in [the home] or maybe a couple of weeks, just building up to the conversation, easing them into it. [Staff focus group, nurse, specialist EMI service 2]

Our staff are not frightened to hold that conversation with relatives, to say ‘look your relative is deteriorating, have you considered’ or, even with the patient themselves ‘have you thought of an advance care plan being put in place’[Interview, service manager, residential/nursing home]

Staff accepted some families were reluctant to talk about EOLC. Strategies to engage families included: identifying a member of staff with a close relationship to the family; identifying the family member who was thought to be most receptive (typically sons or daughters, rather than spouses); and asking a health care professional to talk to the family. One successful approach to engaging families in ACP was via placing the person with dementia’s quality of life central to decisions about future treatment and hospitalisation. Notwithstanding, staff were generally respectful if families were not ready to participate in such discussions.

…you say, ‘we’re not going to make your mum, or your dad, or your husband any better, but we can make sure that they’re very comfortable, that you’re with them when you want to be.’ [Interview, senior manager/director, residential/nursing home]

I think you’ve got to try but then you have to also respect their decision if they’re not ready to do it. [Staff focus group, nurse, specialist EMI service 2]

In summary views on good practice for communicating with families about EOL were consistent across settings and type of staff. However, the timing and scope of initial discussions varied, with some services holding detailed discussions at an early stage, whilst others favoured a more gradual approach.

### Collaborative working

Effective collaborative working underpinned good EOLC for people with dementia and led to: reducing hospital admissions; sharing expertise; building support networks; and negotiating access to services. Key to achieving effective collaborative working was a successful relationship with the GP.

However, access to GPs was variable. The two specialist EMI homes had a planned weekly GP ward round with one specific GP which was highly valued, enabling the GP to get to know the residents, staff and (to a variable extent) families; this facilitated continuity of care and proactive access to clinical expertise: In other services, contact with GPs was arranged as needed with most services dealing with multiple GP practices and GPs. However, challenges arose due to the varying levels of interest and skills held by GPs to provide EOLC for people with dementia:

He [GP] is only allocated an hour […] But he’s very, very supportive, very good. He knows we’re passionate about keeping our residents here […] He respects that, and he supports us as much as he can with medication, with talking to families […] [Staff focus group, nurse, specialist EMI service 1]

We’ve one GP practice we get absolutely superb support and care […]. With another GP practice it’s all instigated by us with very little input from the GP and very little support and very little partnership. [Interview, senior manager/director, residential/nursing home]

GPs sometimes can be, they can be a bit dismissive of us, of our staff reporting concerns and sometimes they can be just a little bit patronising and unaware of the level of empathy and skill amongst our staff […] [Interview, service development lead, supported living]

District nursing involvement in care homes was variable; services with no direct access to in-house nursing support (e.g. home care services or specialist EMI homes staffed by mental health nurses) tended to rely on the district nursing team for practical support. Continual re-organisation of community teams often posed specific challenges. Unsurprisingly, staff found it difficult to keep pace with such changes.

The reshuffling of district nursing teams, and reorganisation of teams, can sometimes can be quite complex and quite difficult. [Staff focus group, nurse, specialist palliative care service 2]

Another problematic area regarding continuity of care was access to out of hours support, particularly at night. There was a perception that contacting the out of hours service may result in (unnecessary) hospitalisation. Some of these difficulties could be avoided by shared documentation, clear communication and developing good relationships with hospitals, which could help ensure that any people with dementia who were admitted would be discharged promptly:

Now we are able to inform them [ambulance crew] as soon as somebody has an emergency health care plan […] and they get a red flag put on the system so if they did receive an emergency call from [nursing home] they already know that there’s a DNR in place… [Interview, service manager, residential/nursing home]

It’s a very lonely place to be, is the care home, particularly at night if you’re the only nurse on and you’ve got to have a very strong clinician who wants to make a decision and sometimes you just need to talk that round with somebody. [Interview, service development lead, specialist palliative care service]

Staff in hospices indicated that they were relatively inexperienced in EOLC in dementia. There was a consensus that hospice-based care was not appropriate for this condition and their role might be in outreach and training. Outreach by hospices was seen as potentially addressing perceived (and not dementia specific) shortcomings of care homes such as: high staff turnover, lack of confidence with managing medical aspects of care (e.g. syringe drivers; medication); and problems in recognising EOL. However, care home service managers interviewed saw themselves and their staff as having expertise in EOLC in dementia, suggesting a potential mismatch between what hospices were trying to deliver (outreach) and what care homes actually needed:

…we are one of the units that has quite a lot of placements for training and we have specialty doctors on, we have specialist registrars on rotation here. I think we could extend that by offering training opportunities to other specialities […] [Interview, doctor, specialist palliative care service]

…we have got the appropriate skills and the appropriate knowledge, we’re able to deliver the support that’s proven to be very effective and secure [the] satisfaction of everybody that’s involved. [Interview, service manager, nursing home]

In summary service managers and frontline staff considered a close network of relationships with other professionals, especially the GP, to be important to ensuring good EOLC; however improved support out of hours was consistently identified as a key area for improvement.

### Continuity of care

Ensuring that care was consistently delivered in a familiar place and with known staff was seen as fundamental. The extent to which service organisation and policies supported continuity varied. In some services an increase in required nursing care could be accommodated without moving the person with dementia, but not in others. Flexibility in staffing for supported living could ensure that people with dementia were able to remain in their own home, with any necessary support. Where possible, staff worked extra shifts or provided cover for their colleagues to ensure continuity of care:

In this sad time the families appreciate that they’re not having a stranger coming in, thinking ‘who is this coming in to deal with my mam’s personal care?’ They know exactly who is coming in [Staff focus group, senior care assistant/team lead, supported living service 1]

Continuity of care was viewed as being undermined by unnecessary hospital admission. Although there were situations in which hospitalisation was appropriate (such as after a serious fall), in general participants felt that hospitals were not a good environment for people with dementia, particularly towards the EOL:

[A person with dementia] had three hospital admissions, he’d had five lots of different antibiotics but all different doctors and when I actually sat down and spoke to the family and said ‘I think he’s dying’ nobody had actually just sat them down and made that decision. [Interview, nurse, primary care]

…it is genuinely distressing because you don’t know anything about the person, their relatives come in at visiting time and you don’t know anything about them, […] I feel it is quite wrong. [Interview, doctor, specialist palliative care service]

There were a number of challenges to avoiding inappropriate hospitalisation, including obtaining medical support out of hours, family preferences for admission, lack of clarity over support, a lack of credible documentation and perceived hierarchies of knowledge/expertise. Involving the out of hours service could result in unwanted admission, particularly where staff lacked confidence to challenge GPs or paramedics. Formal documentation (agreed with families) could help, particularly where preferences were recorded. Even where ACP were in place, problems could arise if they had not been distributed (e.g. to the ambulance service) or their validity was questioned:

That paperwork has made quite a bit of difference. You can actually say to the [paramedics/OOHs GP] ‘You’re not having them. You can treat them, but you’re not having them.’ [Staff focus group, nurse, specialist EMI service 1]

In summary continuity was widely recognised as being key to delivering good EOLC. There was a shared view that the personal needs of people with dementia at EOL are best met by known staff in their usual place of residence. Unnecessary hospitalisation was identified as undermining continuity, but could be managed by agreed, shared documentation and by confident staff able to challenge.

### Ensuring comfort at EOL

The importance of ensuring comfort at the EOL was emphasised by all. Comfort comprised a sense of companionship and compassion in a familiar environment, as well as the absence of pain, attending to hydration and promoting skin integrity. Comfort at EOL was thought to be no different for people with dementia. Identifying and treating pain, through pain assessment, provision of medication in an appropriate form and maintaining the right level of pain relief for prolonged periods, was key. However the use of pain assessment tools was rare, with staff relying on the interpretation of non-verbal signs of discomfort. Care home staff valued working with nurses with training on pain management, often from hospices, and who were proactive and flexible in this area:

A lot of our [residents] […] they’re not able to do tell you if they are in pain or in discomfort or if there’s anything wrong…., that’s where we have to be so really, you know, really observant and spend a lot of time with the person. [Interview, service manager, specialist EMI]

[…] that [hospice based] course has given me the confidence to give pain relief before it gets to a point, I’m not saying I left people in pain but I’m more confident and able to give the medication now […] [Staff focus group, nurse, residential/nursing home 2]

Access to anticipatory medication enabled common symptoms at the end of life such as anxiety, secretions or nausea to be managed promptly. Prescriptions had to be negotiated with GPs or through district nurses and collected. For this reason, planning ahead was seen as vital to avoid ‘rushing around’ at the last minute:

…on a weekend you don’t have your office staff or your maintenance staff and things to be able to drop things and go and sort of fill prescriptions and go and find pieces of equipment that you need. [Interview, service manager, residential/nursing home]

Creating a comfortable environment was also discussed; this could be achieved by encouraging a family atmosphere, configuring lighting, having a radio or music in the background and, if appropriate, having fresh flowers. In addition to a comfortable environment, staff also made sure that basic needs were met (including having favourite foods in an appropriate consistency and providing mouth care).

Along with addressing physical causes of discomfort, companionship at EOL was regarded as a key element of comfort; this could be achieved by allocating a specific member of staff to take responsibility, although this was not always possible. It was felt this was particularly important for people with dementia with no family, reflecting an expressed commitment that people did not die alone. Even if family members were present, they too sometimes required support from staff:

…he [son] didn’t want to be on his own with her, he’s quite nervous […] he put her favourite song on. And I was in the room with them, and he just got her hand and he looked at me and I just walked out the room and they just sat together, and she just slowly passed away. And it was like the perfect end, if there can be a perfect end […] [Staff focus group, care assistant, specialist EMI service 2]

Care after death was mainly discussed by staff providing 24 hour care. For these staff, care did not end with death and the process of preparing the body gave them a sense of satisfaction:

That, that’s what we were doing with [name] when we were washing him, we were just like, “Oh we’re just going to, it’s a little bit cold, don’t worry. We’ll heat it up when we do the rest of you. Just lift your arm up for us.” […] See I don’t like thinking once they’ve passed away they’re just a body. [Staff focus group, nurse, specialist EMI service 2]

In summary, a consistent view of comfort at the EOL was expressed by all participants. Although pain management was seen as a specific issue for people with dementia and limited communication, staff were also attentive to ensuring emotional and environmental factors were addressed.

### Supporting families

Staff from all services recognised the importance of knowing and emotionally supporting families. In some services it was reflected in the provision of carer support plans (documenting support needs, involvement and contact arrangements); in hospices, family support was provided through a daily catch up meeting and /or formal family meetings, enabled by high staff-patient ratios and volunteers. Both hospices and care homes provided open visiting for families, facilitating informal contact. Accommodation or a separate area for family members who wanted to stay was also felt to be important, although not always possible. For people dying in their own homes, support was particularly needed where dying was protracted:

…we have staff to ensure that there is time for the conversations that need to take place at the end of life […] so they can spend a couple of hours with a patient and their family, nobody is clock watching […] [Interview, senior manager/director, specialist palliative care service]

People can sustain looking after somebody at the end of life for a few days but if it goes on it gets very difficult […] families get mentally and physically exhausted unless there’s a big family […] they get stressed, then they fall out with each other and all the tension rises. [Service development lead, supported living]

Staff recognised the need for ongoing support for older, lone relatives/carers. In care homes, this was achieved through continued contact during and after death. In addition to emotional support, staff often offered practical support, such as contacting the undertakers or other family members:

…we would phone the undertaker and we would, not make any arrangements, certainly we couldn’t do that […] the family has been so upset and they don’t feel that they could ring, and start making phone calls to undertakers. [Staff focus group, senior care assistant/team lead, supported living service 1]

While staff generally expressed positive attitudes, in one care home frontline staff were more judgemental, particularly about families who did not visit. In another home, staff reflected on how training had affected their approach:

I mean at one time we used to judge relatives–‘oh, they’re not coming to see them’ and all this kind of thing. And now we think ‘well relatives think in different ways’ and how they [are] with the grief… [Staff focus group, care assistant, residential/nursing home 2]

Providing emotional and practical support to families was viewed as a key aspect of EOLC. Organisational factors (such as accommodation, open visiting, and food and drink) helped families feel welcome and enabled them to spend time with people with dementia. Isolated family members were thought to be particularly vulnerable after bereavement. There was widespread recognition that support did not end when someone with dementia died and typically service managers contacted family members to check whether any additional support was needed.

### Developing and supporting staff

Views about staff training and support highlighted important differences between types of services and staff. The priorities of services were linked to their underlying ethos and values. The ethos of services had an impact on recruitment and management as well as on the care provided. Values-based services were less concerned with experience and qualifications of new staff; the emphasis was on the qualities required to fit well with the ethos. Several services commented on the need for experienced staff to embrace new methods. One home preferred ‘naïve’ staff who had strong caring and empathetic qualities. Approaches and commitment to the provision of training similarly varied. Some external professionals perceived care homes as unwilling to fund even inexpensive training and questioned the benefit due to staff turnover. Staff across all services valued training in technical skills; for nurses this included pain management and use of syringe drivers, for care staff learning how to wash and dress someone after death. The issue of maintaining skills through coaching was raised by staff, rather than relying on other services to set up equipment such as syringe drivers:

They [staff] are trained extremely well […] it’s not just the mandatory training, they have a lot of other background knowledge with dementia etc. […] and it’s always updated. It’s not a one-off, it’s constantly given, they could have two of three training days a month on different things. [Staff focus group, senior care assistant/team lead, residential/nursing home 2]

[distance learning] you had the domestic staff sat around beside healthcare, they’re sat around trained nurses and then you know you’re all hearing the same message, and getting a feel for how it fits in with your role. [Interview, nurse, residential/nursing home]

Emotional attachment to people in their care was predominantly discussed by frontline staff rather than senior managers. Some managers felt staff should be compassionate and able to show emotions without “fear of being belittled”; others thought staff should remain detached. For most frontline staff, attachment to the people they care for was inevitable; delivering EOLC especially had an emotional impact which highlighted the need for staff support.

…families are so appreciative, because it’s not like a big residential home, it’s a small unit and there’s only four people living there. So you get closely involved [Staff focus group, senior care assistant/team lead, supported living service 1]

A sense of satisfaction for staff was key to offsetting feelings of loss. Several services held a post-death review meeting to focus on what went well but also to process emotions and identify training needs. Often after death there was a reliance on staff members accessing informal support, fostered by working in close teams and supplemented by structured meetings (including debriefing after a death, handover meetings and supervision). Some staff described how they would reminisce, which seemed effective in lightening the mood and processing the emotional impact:

We talk about the significant events that happened, i.e. if we had a couple of deaths, we review those deaths, how did they happen, what did we do well, what could we have done better, if there was any learning that we could bring from that person. [Interview, service manager, specialist EMI]

…with a lot of the residents that have passed away recently, names still get brought up of stories and history. [Staff focus group, nurse, specialist EMI service 2]

Almost half of the service managers based in hospices commented on the lack of knowledge or interest in dementia among their own staff. A common theme was ‘fear’ of people with dementia, both individually and in compromising their existing focus. Despite these issues, hospices were keen to offer support and identified care homes as needing their input. There seemed little recognition that existing services might have their own expertise in managing dementia which could be of value to hospices:

You have a lot of staff who really, really struggle to understand dementia, understand the relevance of life history, […] who just can’t begin to understand how to deal with behaviours that might challenge and unpick that… [Interview, service development lead, specialist palliative care service]

It feels like we need to be going in much more proactively and saying, “Look, you know, actually the vast majority of your residents are approaching the end of life, how can we help? […] we’re actually aware they’re going to learn much more from discussion, working alongside, that kind of experiential learning. [Staff focus group, doctor, specialist palliative care service 2]

Differences in the values and ethos of individual services had wide reaching effects on the degree of training and support provided to staff. In some services, the concept of person centred care informed not only their approach to people with dementia, but also encompassed families and staff. In particular, it influenced attachments between staff and people with dementia and the extent to which the emotional aspects of EOLC were recognised and supported.

## Discussion

Service managers and frontline staff identified seven key themes important to the provision of good quality EOLC for people with dementia and their families: these were recognition of the need for EOL and use of appropriate EOLC tools; communicating appropriately with families; collaborative working; continuity of care; ensuring comfort; supporting families; and developing and supporting care staff. Findings from such qualitative research can help inform the development of a complex evidence-based intervention to support professional carers to deliver better quality EOLC in dementia [49]. Our findings, grounded in in-depth empirical research resonate with key research priorities identified in a recent European expert consensus initiative to generate guidelines for optimal EOLC in dementia [23]. These include: person-centred care, communication and shared decision-making; optimal treatment of symptoms and provision of comfort; and advance planning.

Recognition of the EOL phase in dementia is acknowledged to be very difficult due to the uncertainty and unpredictability of the dying trajectory [50]. Participants identified two aspects of essential knowledge to help this: i) clinical knowledge of the dying process in general and ii) detailed personal knowledge about the person with dementia in terms of their personality, personal history and usual behaviour. Combination of both helped facilitate recognition of the physical, emotional and verbal cues to signal a more advanced stage of the illness. While much of this expertise came from experiential learning, having organisational systems and processes in place also helped for example, case review meetings, use of clinical care pathways and advance care planning discussions and completion of written forms such as emergency health care plans and Do Not Attempt Resuscitation documents [41]. Although clinical fluctuations were expected by staff, they still found instigating the timing of ACP conversations challenging; this mirrors findings from previous research exploring how best to implement ACP in dementia care [44, 50]. More successful ACP communication with families may be achieved through: using both abstract (talking about planning for death in general) and more specific conversations (specific individual issues the family need to make decisions on); embedding EOL information in routine meetings with relatives; conceptualising EOLC in terms of supporting the well-being and comfort of people with dementia, and having the member of staff most familiar with the person and their family initiate discussions.

The degree of collaborative and co-ordinated working varied across services. Continuity was viewed as important, with inappropriate hospitalisation the principal threat to continuity of care; participants felt it was crucial that people with dementia remain in their usual place of care for as long as possible [11, 51]. Having the flexibility for people with dementia at EOL to remain under the care of the same staff went some way to easing the heightened emotions of family members. Out of hours support was consistently identified as an area for improvement, with poor provision often contributing to unnecessary hospitalisation. It has been suggested that integration of nursing homes into EOLC networks might help improve care [52], especially out of hours care, and ensure better collaboration with palliative care services for expert support [53]. However within palliative care services there were some negative perceptions about i) the ability of care home staff to deliver good quality EOLC and ii) about the role of hospices in caring for people with advanced dementia. This may be explained by a lack of knowledge and expertise in palliative care teams about dementia care and concern regarding the ability of hospices and palliative care teams to cope with this increased burden [11, 54, 55]. Staff in care homes often have valuable expertise in providing dementia which should be recognised by palliative specialists. One solution to these issues is the establishment of community dementia palliative care teams with specialist nurses adopting a lead co-ordinator role [56]. For successful collaborative working, our participants identified GPs as key; their active engagement in EOLC for people with dementia was highly valued by care home staff. Interestingly there was a recurring perception in care home staff that failures in care were as a result of other professionals’ actions. More formal opportunities for these staff to engage in inter-disciplinary learning and reflective practice should be encouraged (this is in marked contrast to postgraduate medical training in the UK where annual appraisal and reflective practice is mandatory), though staff retention and organisation stability is key to success [57]. Policy initiatives such as the National Dementia Strategy are seeking to improve practice in this area [19].

With regard to the specific nature of care provided, expert consensus has placed symptom management and comfort as a top priority [23]. Comfort was emphasised by all staff participants, with the assessment and treatment of pain an important area. Planning for the use of anticipatory medicines was considered essential but creating a comfortable environment–incorporating favourite foods, mouth care and companionship–was viewed as equally important. Recent national guidance on EOLC strongly highlights the need to address all of these key areas to maintain comfort and quality of life [21, 22]. Notwithstanding, our participants also identified the need to provide emotional support for families as a core aspect of good EOLC practice [58, 59] as emotions can have a major influence on treatment decisions for people with dementia at EOL [60]. The degree of staff involvement depends on an individual’s approach to patient/family engagement and their abilities and experience in engaging in this area [29], but also the underlying values and ethos of the care home service. Having staff in post who have personal qualities which enable them to develop (emotional) relationships with families and close-working with immediate colleagues reflected a person-centred ethos. In these situations post-death reviews were a valuable space to both reflect on practice, and the need for additional training, but also to allow staff to process the deep personal emotions they often felt having cared for a person with dementia for some considerable time.

The study had some limitations. Our sample–produced by convenience and purposive sampling–covered a variety of service types, staff roles and a relatively large number of participants. However, it is possible that by focusing on sites of good and standard practice we have underrepresented services with sub-standard approaches. Recruiting managers and staff from such services is difficult owing to a reluctance to discuss poor care. By focusing on good and standard practice we aimed to provide evidence on how good EOLC for people with dementia can be delivered and the form it should take. Also, the study draws on a relatively large qualitative dataset comprising the views of a variety of professionals. Amore specific limitation relates to the presence of service managers in focus groups. Although we clarified focus groups were aimed at frontline staff, in two focus groups service managers were present (a practical concession to ensure the focus groups took place). It is possible that this altered the dynamic between participants.

## Conclusions

In conclusion, we identified seven key areas which staff consider are crucial to the provision of good quality EOLC in dementia. A major implication of this study is the need to recognise, value and better support the expertise of nursing and care home staff in this area; this should come from both within the ‘organisation’ (by developing an ethos which values both the physical ‘hands on’ and emotional work needed in providing such cares) and from better collaborative working with healthcare services including GPs and palliative care teams. Better quality care can be achieved by ensuring continuity of care (for example named lead care home nurse and lead GP; planned OOH care), earlier more timely discussions with both people with dementia and their families to understand their personal wishes and appropriate documentation and dissemination of relevant ACP forms and closer, more collaborative working with specialist palliative care services, who provide both essential clinical advice but also support and mentor less qualified staff. A key component of good quality EOLC in dementia is also successful engagement with, and support of, family members both before and after the person with dementia’s death. Future research should explore how best this can be achieved within current service configuration and organisation and if new service initiatives, such as EOLC networks or community dementia palliative care nurses, are required above and beyond usual care to support professionals to deliver good quality care towards, and at, end of life in dementia.

## Supporting information

S1 Checklist COREQ checklist. (DOCX)

## Abbreviations

ACP: Advance care planning
DNACPR: Do Not Attempt Cardiopulmonary Resuscitation
EMI: Elderly mentally infirm
EOL: End of life
EOLC: End of life care
GSF: Gold Standards Framework
LCP: Liverpool Care Pathway
MCA: Mental Capacity Act
