# Supplementary material for: End of life care for people with dementia: The views of health professionals, social care service managers and frontline staff on key requirements for good practice
Source: PLoS One. 2017 Jun 16;12(6):e0179355. doi: 10.1371/journal.pone.0179355 (PMC5473529; doi:10.1371/journal.pone.0179355)
Supplement: S1 Checklist — (DOCX) [file pone.0179355.s001.docx]

Domain 1: Research team and reflexivity

Personal Characteristics

1. Interviewer/facilitator Which author/s conducted the interview or focus group?

RPL, MP, CB, EMc

2. Credentials What were the researcher’s credentials? E.g. PhD, MD

RPL (PhD), MP (MSc), CB (MSc), EMc (MSc)

3. Occupation What was their occupation at the time of the study?

RPL (Researcher), MP (Researcher), CB (Researcher), EMc (Researcher)

4. Gender Was the researcher male or female?

RPL (male), MP (female), CB (female), EMc (female)

5. Experience and training What experience or training did the researcher have?

Experienced qualitative researchers who have received training in consent and capacity issues and GCP training

Relationship with participants

6. Relationship established Was a relationship established prior to study commencement?

Team had existing knowledge of some service managers interviewed, but relationships were mainly developed during the study

7. Participant knowledge of the interviewer What did the participants know about the researcher? e.g. personal goals, reasons for doing the research

Participants received a ‘participant information sheet’ and the researchers explained the study before commencing the interview or focus group

8. Interviewer characteristics What characteristics were reported about the interviewer/facilitator? e.g. Bias, assumptions, reasons and interests in the research topic

Researchers discussed their interest in services recognised for having good practice

Domain 2: study design

Theoretical framework

9. Methodological orientation and Theory What methodological orientation was stated to underpin the study? e.g. grounded theory, discourse analysis, ethnography, phenomenology, content analysis

Interpretive approach and thematic analysis

Participant selection

10. Sampling How were participants selected? e.g. purposive, convenience, consecutive, snowball

Combination of purposive and snowball sampling

11. Method of approach How were participants approached? e.g. face-to-face, telephone, mail, email

Combination of telephone, email and face-to-face invitations

12. Sample size How many participants were in the study?

87

13. Non-participation How many people refused to participate or dropped out? Reasons?

No participants refused or dropped out after being approached. However some service managers contacted did not reply to our approaches.

Setting

14. Setting of data collection Where was the data collected? e.g. home, clinic, workplace

Telephone interviews and in the service where service managers and staff worked

15. Presence of non-participants Was anyone else present besides the participants and researchers?

No

16. Description of sample What are the important characteristics of the sample? e.g. demographic data, date

The important characteristics of the sample are their roles in the service they worked in and the type of service.

Data collection

17. Interview guide Were questions, prompts, guides provided by the authors? Was it pilot tested?

There was a semi-structured interview guide and a focus group topic guide

18. Repeat interviews Were repeat interviews carried out? If yes, how many?

No

19. Audio/visual recording Did the research use audio or visual recording to collect the data?

Yes, audio recording and full transcription

20. Field notes Were field notes made during and/or after the interview or focus group?

Yes, in some cases where relevant

21. Duration What was the duration of the interviews or focus group?

The interviews varied in length from 27mins to 1h 03mins. Focus groups varied in length from 48mins to 1h 48mins.

22. Data saturation Was data saturation discussed?

Yes, during our on-going analysis.

23. Transcripts returned Were transcripts returned to participants for comment and/or correction?

No

Domain 3: analysis and findings

Data analysis

24. Number of data coders How many data coders coded the data?

4

25. Description of the coding tree Did authors provide a description of the coding tree?

No

26. Derivation of themes Were themes identified in advance or derived from the data?

Derived from the data

27. Software What software, if applicable, was used to manage the data?

NVivo

28. Participant checking Did participants provide feedback on the findings?

No

Reporting

29. Quotations presented Were participant quotations presented to illustrate the themes / findings? Was each quotation identified? e.g. participant number

Yes

30. Data and findings consistent Was there consistency between the data presented and the findings?

Yes

31. Clarity of major themes Were major themes clearly presented in the findings?

Yes

32. Clarity of minor themes Is there a description of diverse cases or discussion of minor themes?

No
